# Cognitive insight in psychosis: The relationship between self-certainty and self-reflection dimensions and neuropsychological measures

**DOI:** 10.1016/j.psychres.2009.05.009

**Published:** 2010-07-30

**Authors:** Michael A. Cooke, Emmanuelle R. Peters, Dominic Fannon, Ingrid Aasen, Elizabeth Kuipers, Veena Kumari

**Affiliations:** a Department of Psychology, Institute of Psychiatry, King's College London, London, UK; b Division of Psychological Medicine and Psychiatry, Institute of Psychiatry, King's College London, London, UK; c NIHR Biomedical Research Centre for Mental Health, South London and Maudsley Foundation NHS Trust, London, UK

**Keywords:** Insight, Schizophrenia, Executive functioning, Introspection, Distorted beliefs, Resistance to correction

## Abstract

Cognitive insight in schizophrenia encompasses the evaluation and reinterpretation of distorted beliefs and appraisals. We investigated the neuropsychological basis of cognitive insight in psychosis. It was predicted that, like clinical insight, cognitive insight would be associated with a wide range of neuropsychological functions, but would be most strongly associated with measures of executive function. Sixty-five outpatients with schizophrenia or schizoaffective disorder were assessed on tests of intelligence quotient (IQ), executive function, verbal fluency, attention and memory, and completed the Beck Cognitive Insight Scale, which includes two subscales, self-certainty and self-reflection. Higher self-certainty scores reflect greater certainty about being right and more resistant to correction (poor insight), while higher self-reflection scores indicate the expression of introspection and the willingness to acknowledge fallibility (good insight). The self-certainty dimension of poor cognitive insight was significantly associated with lower scores on the Behavioural Assessment of Dysexecutive Syndrome; this relationship was not mediated by IQ. There were no relationships between self-reflection and any neuropsychological measures. We conclude that greater self-certainty (poor cognitive insight) is modestly associated with poorer executive function in psychotic individuals; self-reflection has no association with executive function. The self-certainty and self-reflection dimensions of cognitive insight have differential correlates, and probably different mechanisms, in psychosis.

## 1 Introduction

Traditional ‘insight’ in psychiatry is most commonly viewed as a multi-dimensional construct incorporating awareness of illness, symptoms and the need for treatment (David, 1990; Amador et al., 1991). Individuals diagnosed with schizophrenia frequently disagree with mental health professionals about the nature of their experiences, and whether they are in need of psychiatric treatment such as medication. Such disagreements are generally viewed as reflecting poor insight on the part of the patient in one or more of these dimensions, which in turn has been linked to poor medication compliance and then to poor outcome (Morgan and David, 2004). There is a more recent suggestion that good insight can have maladaptive consequences for self-esteem and causes distress (Cooke et al., 2007a,b).

The neuropsychological correlates of traditional insight in schizophrenia have been investigated extensively. While there has been considerable heterogeneity in the results of these studies (Cooke et al., 2005), a recent meta-analysis (Aleman et al., 2006) has shown that poor insight is associated with poor functioning in a range of cognitive domains, including intelligence quotient (IQ), memory and executive function. There is also some evidence to suggest that the associations are particularly strong for the set-shifting and error monitoring aspects of executive function (Aleman et al., 2006).

Recently, Beck and colleagues (Beck and Warman, 2004; Beck et al., 2004) have distinguished between the traditional approach to insight, which they term ‘clinical insight’, and ‘cognitive insight’, which is a form of cognitive flexibility and encompasses the evaluation and correction of distorted beliefs and misinterpretations. They contend that a crucial cognitive problem in the psychoses (including schizophrenia) is that individuals are unable to distance themselves from their cognitive distortions (e.g., ‘there is a conspiracy against me’), and are also impervious to corrective feedback (Moritz and Woodward, 2006). In contrast, individuals with panic disorder or obsessive–compulsive disorder are more likely to retain the ability to recognise that the conclusions they have made were incorrect, and therefore maintain good cognitive insight.

A lack of cognitive insight in individuals with schizophrenia contributes to both the impairment of clinical insight, and the development of delusions (Beck and Warman, 2004). An impairment in the capacity to evaluate misinterpretations and alter appraisals despite feedback from others, may lead an individual with schizophrenia to disagree with others who call their experiences symptoms of illness; this disagreement is then called an impairment of the ‘awareness of symptoms’ aspect of clinical insight. Poor cognitive insight, it is then argued, may also lead such individuals to conclude that their interpretations (appraisals) of their experiences (e.g., ‘there is a conspiracy against me’) are factually correct (Beck et al., 1994), contributing to the formation and maintenance of delusions. As in other cognitive models, cognitive models of psychosis emphasise the particular role of appraisals in delusional formation and maintenance (Garety et al., 2007). Recent findings have highlighted the potential importance of cognitive insight as a mediator of response to cognitive behavioural therapy for psychosis (Granholm et al., 2005), with increases in cognitive insight associated with reductions in positive, negative and general symptomatology (Granholm et al., 2006).

The Beck Cognitive Insight Scale (BCIS; Beck et al., 1994) has two distinct subscales, self-certainty and self-reflection. Poor insight is characterised by a high degree of certainty in one's (mis)interpretations, and a lack of self-reflectiveness. Beck and his colleagues (Beck and Warman, 2004; Beck et al., 2008) suggest that the BCIS is an indirect test of a putative impairment of the ‘higher level’ functions in schizophrenia, with the process of distancing oneself from highly salient (delusional) beliefs and having the capacity to view them in perspective requiring intact executive function.

The objective of this study was to examine the neuropsychological correlates of both dimensions of cognitive insight in schizophrenia. We hypothesised that, like clinical insight, cognitive insight will be associated with a wide range of neuropsychological functions, but will be most strongly associated with measures of executive function, particularly measures of set-shifting and error monitoring. These aspects of executive function, as stated earlier, have been found to show most strong associations with clinical insight (Aleman et al., 2006).

## 2 Method

### 2.1 Participants

Sixty-five outpatients (46 men, 19 women) who met the *Diagnostic and Statistical Manual of Mental Disorders*, fourth edition (DSM-IV) (American Psychiatric Association, 1994) criteria for the diagnosis of schizophrenia or schizoaffective disorder were recruited from the South London and Maudsley Foundation NHS Trust. The mean age of the participants was 38.9 years (range 19–65). All participants were on stable doses of antipsychotic medication (51 on atypical and 14 on typical antipsychotics) for at least 3 months prior to taking part in this study, were in a stable (chronic) phase of illness and were recruited from the community (all outpatients). The mean ratings of the participants on the Positive and Negative Syndrome Scale (PANSS; Kay et al., 1987) were 16.8 (S.D. = 4.9) for the positive subscale, 18.3 (S.D. = 5.0) for the negative subscale and 32.5 (S.D. = 6.5) for the general psychopathology subscale. The study sample was the same as in Cooke et al. (2007a; this report did not examine neuropsychological data in relation to the BCIS or any other measure of insight).

After complete description of the study to the participants, written informed consent was obtained. The study procedures were approved by the Joint Research Ethics Committee of the South London and Maudsley NHS Foundation Trust and Institute of Psychiatry.

### 2.2 Clinical measures

Diagnoses were ascertained by a consultant level psychiatrist (DF) using the Structured Clinical Interview for DSM-IV Axis I disorders Research Version (SCID-P; Spitzer et al., 1994), who also administered the PANSS (Kay et al., 1987) and was blind to both the cognitive insight and neuropsychological test scores.

Cognitive insight was assessed using the BCIS (Beck et al., 1994), a 15-item self-report scale which measures the two dimensions of self-certainty and self-reflectiveness (but not insight into potential neuropsychological functioning deficits). Items are rated by the participant on a four-point scale from ‘do not agree’ to ‘agree completely’. The self-certainty dimension is calculated as the sum of six items (possible range 0 – 18), and measures decision making regarding mental products: certainty about being right and resistance to correction (Beck et al., 1994), for example ‘I know better than anyone else what my problems are’. Greater self-certainty indicates poorer cognitive insight (i.e., overconfidence in decision making). The self-reflectiveness dimension is calculated as the sum of the remaining nine items (possible range 0 – 27) and measures the expression of introspection and willingness to acknowledge fallibility (Beck et al., 1994), for example ‘If someone points out that my beliefs are wrong I am willing to consider it’, with a higher score indicating better cognitive insight.

The G12 item of the PANSS (‘lack of judgement and insight’) was collected as part of PANSS assessment and was subsequently used to assess the convergent validity of the cognitive insight measures. Clinical insight in this patient sample was also assessed using the Birchwood insight scale (BIS; Birchwood et al., 1994) and the expanded Schedule of Assessment of Insight (SAI-E, Kemp and David, 1997). Both of these measures assess David's (1990) three dimensions of clinical insight, namely (i) the presence of a mental illness, (ii) the need for treatment and (iii) the identification of symptoms as abnormal. The SAI-E also includes an additional item on awareness of the social consequences of illness. When administering the BIS, question 4 (“My stay in hospital is necessary”) was excluded because all patients of this study were outpatients. The remaining three items from the ‘awareness of the need for treatment’ dimension were used to calculate a score for this subscale with equal weight to the other two subscales, allowing a total score to be calculated which has the same range (0–12) as the full BIS Scale. Questions 7 and 8 of the SAE-I (which assess the level of insight into the presence of symptoms) were excluded for eight patients, as they did not possess any symptoms for which insight could be rated. Higher scores indicate better insight on both the BIS and SAI-E.

### 2.3 Neuropsychological measures

General intellectual ability was assessed using the full-scale IQ estimate derived from the two-subtest version of the Wechsler Abbreviated Scale of Intelligence (WASI; Wechsler, 1999), which consists of the Vocabulary and Matrix Reasoning subtests.

Executive function is a diverse construct, which encompasses a large number of processes (Godefroy, 2003). A broad battery of executive function tests was therefore employed to capture this diversity. The tests used (followed by the dependent variables) were the Wisconsin Card Sorting Test (WCST, computerised version; Heaton et al., 1993) – categories completed, % perseverative errors and % non-perseverative errors, the Trail Making Test (TMT; Reitan, 1955) – ‘difference score’ (part B time minus part A time, which controls for psychomotor speed), Brixton Spatial Anticipation test (Burgess and Shallice, 1997) – profile score, Hayling Sentence Completion test (Burgess and Shallice, 1997) – profile score, the Behavioural Assessment of Dysexecutive Syndrome (BADS; Wilson et al., 1996, 1998) – total score across the six subtests and the Stroop Colour-Word test (Golden, 1978) – interference score.

Explicit declarative memory (total recall) was assessed using the Hopkins Verbal Learning Test (HVLT; Shapiro et al., 1999).

Verbal fluency was assessed using the Controlled Oral Word Association Test (COWAT; Milner, 1975), which measures both phonological and semantic fluency using letter and category verbal fluency tests respectively. The total number of correct responses for the three letter fluency conditions was used as the measure of phonological fluency, while the total number of correct responses for the three category fluency conditions was used as the measure of semantic fluency.

The computerised Continuous Performance Test, Identical Pairs Version (CPT-IP; Cornblatt et al., 1988) was used to assess sustained attention. Performance on the CPT-IP task is indexed by ‘hits’ (responses to match trials) and false alarms (responses to catch trials). These two scores yield the signal detection index ‘d'Prime’, which represents the signal-to-noise ratio by measuring the sensitivity of the participant to the discrimination of targets from catch trials.

### 2.4 Statistical analyses

The distributions of all measures were inspected using histograms and *Q*–*Q* plots to determine whether they approximated normal distributions. Non-parametric statistics were applied to variables which did not approximate normal distributions. Two-tailed Pearson's correlations were used to examine relationships between variables which approximated normal distributions, while Spearman's rank correlations were used when one of the variables was not normally distributed. The Bonferroni method was used to control for multiple comparisons, with the standard alpha of *P* < 0.05 divided by the number of tests undertaken for each insight variable, to yield a corrected alpha of *P* < 0.00357.

Following a significant correlation between self-certainty subscale of the BCIS and the BADS total score at a level which survived correction for multiple comparisons, a forward Wald logistic regression with all cognitive measures entered as potential predictors and this subscale of the BCIS as the dependent variable was undertaken to examine the extent to which neuropsychological variables explained separate variance in self-certainty scores. A linear regression was used to determine whether the relationship between self-certainty and BADS total score was mediated entirely by general cognitive ability.

In secondary analyses, we also examined the correlations between insight as assessed by the PANSS G12 item, the BIS and the SAI-E, mainly with a view to replicate previous findings linking poor executive function to poor insight (Aleman et al., 2006).

## 3 Results

### 3.1 Data inspection

The three WCST variables, the Stroop interference score and the Trails B-A time were not normally distributed. The two BCIS scores, the PANSS G12 insight item and the remaining neuropsychological measures approximated normal distributions.

### 3.2 Insight variables

The descriptive statistics for insight measures and the correlations between cognitive and clinical insight measures are presented in Table 1. The PANSS G12 insight item was modestly but significantly correlated with both BCIS dimensions: positively for self-certainty and negatively for self-reflectiveness. Both correlations were in the expected direction, with higher PANSS G12 item scores (poorer clinical insight) associated with scores indicating poorer cognitive insight (higher self-certainty and lower self-reflectiveness). Both BCIS subscales were also significantly correlated with the total scores of the SAI-E and BIS measures of clinical insight in the expected directions (all *r* > ± 0.4, *P* ≤ 0.001) though the strength of the correlations varied for the two subscales and appears somewhat stronger for the BCIS self-certainty scale. No insight measures were significantly correlated with either duration of illness or age of onset (all *P* > 0.1).

### 3.3 Associations between insight and neuropsychological functioning

The descriptive statistics for neuropsychological variables are presented in Table 2. The correlations between the two BCIS dimensions and the neuropsychological test scores are set out in Table 3.

**Fig. 1:**
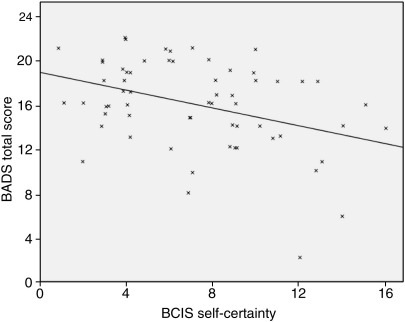
Relationship between BADS total score and BCIS self-certainty.

The self-certainty BCIS dimension was significantly negatively correlated with the BADS total score at a level which survived correction for multiple comparisons (*P* < 0.003). This relationship is presented in Fig. 1. This association was in the expected direction of poorer cognitive insight (higher self-certainty) being associated with poorer neuropsychological functioning. The self-certainty BCIS dimension was also negatively correlated with Brixton test profile score at the single test level (*P* < 0.05), but this did not survive correction for multiple comparisons.

There were no significant relationships between the self-reflectiveness dimension and any of the neuropsychological measures. The strength of the self-certainty–BADS correlation (*r* = − 0.375) was compared to that of the self-reflectiveness–BADS correlation (*r* = 0.020) using Fisher's *Z* transformations. It was found that they were significantly different (*Z* = 2.25, *P* = 0.024).

As the BADS is composed of six subtests, exploratory *post hoc* correlations were undertaken to determine which subtest was most strongly associated with self-certainty. These analyses indicated that the only BADS subtest to show a significant relationship with self-certainty was the modified six elements test (rho = − 0.335, *P* < 0.01). Specifically, it was the number of times that the rules were broken (up to the maximum score of 3 recorded in the scoring key) which were associated with self-certainty score (rho = − 0.332, *P* < 0.01), rather than the number of tasks attempted (rho = − 0.060, ns). This suggests that it was not perseveration on tasks which was associated with high self-certainty, but rather the inability to follow the rules of the task.

In order to determine the extent to which the BADS explains unique variance in self-certainty scores, a logistic regression was undertaken with all neuropsychological variables entered as potential predictors. As expected, BADS total score was the most significant predictor (*P* < 0.01), explaining 10.5% of the variance on its own. WCST % non-perseverative errors also entered the equation as a significant predictor (*P* < 0.05), explaining an additional 8.2% of the variance (total 18.7% variance explained).

In order to determine whether the significant relationships between self-certainty and executive function could be explained in terms of general cognitive ability, a regression was computed where current IQ was entered first, followed by BCIS self-certainty score. The first model was significant, indicating a positive relationship between BADS total score and WASI IQ (*R*^2^ = 0.478, *P* < 0.001). The addition of self-certainty in the second model improved the model significantly (*R*^2^ change = 0.065, *F* change = 8.166, *P* = 0.006) indicating that the relationship between self-certainty and BADS score was not entirely mediated by IQ.

While examining the relationships between clinical insight as assessed by the PANSS G12 item and neuropsychological functioning, a relationship between lower BADS total score and poorer clinical insight was found (*P* < 0.02), as was a relationship between poorer clinical insight and lower verbal fluency (FAS score, *P* < 0.05) (see Table 3). Relationships were also found between lower insight on the illness into symptoms dimension of the BIS and poorer performance on three executive function measures (Hayling profile score, *r* = 0.266, *P* = 0.03; Brixton profile score, *r* = 0.264, *P* = 0.03; BADS total score, *r* = 0.256, *P* = 0.04), and between lower insight on the insight into illness dimension and lower Brixton test profile scores (*P* = 0.292, *P* = 0.02); other BIS dimensions were not correlated with any neuropsychological variables. However, none of the clinical insight–neuropsychological associations survived correction for multiple comparisons. The dimensions of insight on the SAI-E as well as the factors derived from the factor analysis of the BIS and the SAI-E items (as described in Cooke et al., 2007a) did not correlate significantly with any neuropsychological variables.

## 4 Discussion

Cognitive insight has recently emerged as an important aspect of research on insight in schizophrenia, in part due to data suggesting that it is a moderator of how individuals respond to cognitive behaviour therapy for schizophrenia (Granholm et al., 2005, 2006). This study set out to investigate the neuropsychological correlates of the BCIS, the only measure of cognitive insight currently available. Significant relationships were found with two of the six tests of executive function. Significant correlations were found between lower scores on the BCIS self-certainty dimension and higher scores on both the Brixton spatial anticipation test and the BADS; the latter survived Bonferroni correction for multiple comparisons and remained significant after controlling for IQ. Lower scores on this BCIS dimension indicate better decision making regarding mental products – i.e., less overconfidence about the reality of one's experiences, and lower resistance to correction. The relationships found therefore indicate a modest association between higher cognitive insight and better executive functioning, in line with unpublished data (Grant and Beck) cited by Beck et al. (2008).

Probing further into the self-certainty–executive function relationship with *post hoc* analyses showed that, of the six BADS subtests, the relationship with self-certainty was strongest for the ‘modified six elements’ subtest. This subtest is a simplified version of the original Shallice and Burgess (1991) six elements test, and involves participants following instructions to do three tasks (dictation, arithmetic and picture naming), each of which is divided into two parts. The participant is required to attempt at least something from each of the six subtasks within a 10-min period. In addition, participants are not allowed to do the two parts of the same task consecutively. Marks are awarded for the number of tasks attempted in 10 min, and deducted when the rules of the task are broken. The *post hoc* analyses revealed that it was the number of rule-breaks, not the number of tasks attempted, which was associated with the self-certainty score. It therefore appears that poor cognitive insight is associated with the inability to form and follow a strategy, rather than the tendency to perseverate on a particular element of the test.

Poor cognitive insight was not associated with another measure of perseveration, the proportion of perseverative errors on the WCST. Perseverative errors arise through a failure to recognise that a previously successful strategy is now producing errors – i.e., poor error monitoring. This finding is important in the context of a neuropsychological model of poor clinical insight, as it has been suggested that a failure to detect errors may be particularly important in the apparent unawareness of the ‘incorrectness’ of symptoms in individuals with schizophrenia (Larøi et al., 2000). If the same was true of the neuropsychological basis of cognitive insight, one would expect poor cognitive insight to be associated with greater perseveration, which was not the case in this study, thus casting doubt on Larøi and colleague's suggestion. The scores on the other three putative tests of executive function employed in this study, namely, the Stroop test, which primarily assesses selective attention, the Trail Making test, which indexes mental flexibility and the Hayling Sentence Completion test which measures response initiation and suppression, were also not correlated with self-certainty. It is unlikely that this was due to a floor effect, or a limited range of scores on these measures (see Table 2). It therefore appears that the relationship between the self-certainty dimension of cognitive insight and executive function is not driven by a specific cognitive process such as perseveration, but rather is related to the general ability to form and maintain a strategy for problem solving. This is also suggested by the finding of the regression analysis showing that non-perseverative, rather than perseverative, errors explained additional variance (to that explained by the BADS scores) in self-certainty dimension of cognitive insight. It is important to point out that, although increased perseverative errors are often cited as the most characteristic aspect of the performance of individuals with schizophrenia on WCST (Koren et al., 1998), the results of a meta-analytic review suggest that both perseverative and non-perseverative type errors are committed on this test by patients with schizophrenia (Li, 2004).

No relationships were found between self-certainty scores and the other cognitive domains investigated, namely general intellectual ability, verbal fluency, sustained attention and memory. This supports a degree of specificity in the relationship between executive function and the components of poor decision making regarding mental products which make up the self-certainty dimension of cognitive insight: jumping to conclusions, overconfidence about being right and resistance to correction. Recent data suggest that general intellectual ability is significantly associated with the ‘jumping to conclusions’ (JTC) bias (Van Dael et al., 2006), although this study did not measure executive function specifically. Further, JTC, investigated in depth by Garety et al. (2005), is one of the components of cognitive insight that is designed to be measured through the self-certainty dimension of the BCIS. Our findings suggest that poor executive functioning may contribute more strongly than general cognitive ability to individuals with schizophrenia making over-confident decisions.

Self-reflectiveness, the expression of introspection and the willingness to acknowledge fallibility (Beck et al., 1994), was not significantly associated with any neuropsychological measure included in this study. Specifically, the correlations between BADS-self-certainty and BADS-self-reflectiveness were significantly different from each other, suggesting a neuropsychologically different pathway between the two dimensions of cognitive insight.

Other recent data also point to the relative independence of the self-certainty and self-reflectiveness dimensions of cognitive insight. Specifically, higher self-certainty (lower insight), and also higher self-reflection (higher insight), has been observed in delusion-prone college students (Warman and Martin, 2006; delusion-proneness assessed with the Peters Delusion Inventory, PDI; Peters et al., 1999). More recently, psychotic individuals with active delusions have been reported to show lower insight on the self-certainty dimension (over-confident) but greater insight on the self-reflection dimension than those without active delusions (Warman et al., 2007). Taken together, these and our findings suggest that self-certainty and self-reflection are independent dimensions at both the clinical and the neuropsychological levels. However, while delusion-proneness or presence of delusions in studies by Warman and colleagues (Warman and Martin, 2006; Warman et al., 2007) related to both greater self-certainty (poor insight) and greater self-reflection (good insight), poor executive function significantly related only to poor insight on the self-certainty dimension in our study.

Clinical insight, assessed on the scales which are modestly correlated with cognitive insight in our and other data (e.g., Pedrelli et al., 2004), also showed positive associations with scores on some tests of executive function in this study. This is consistent with previous data on this topic (Aleman et al., 2006) and may suggest that self-certainty dimension of the BCIS, despite measuring aspects of insight that differ considerably from clinical insight measures based around the medical model, has similar neuropsychological correlates to that observed previously for clinical insight measures. Although the strength of the correlation between symptoms insight on the BIS and the BADS profile score was somewhat lower, it was not significantly different from that observed for the self-certainty dimension of the BCIS.

Lastly, we found no association between the duration of illness and self-certainty or self-reflectiveness dimensions. In a longitudinal study, Bora et al. (2007) observed lower insight on both self-certainty and self-reflectiveness dimensions in acutely ill psychotic patients and reported some improvement in self-certainty but no change in the self-relectiveness after recovery of psychosis. Our findings seem to suggest that both overconfidence in judgements and impaired self-reflectiveness are fairly stable during the chronic course of the illness (Garety et al., 2007; Warman and Martin, 2006).

This study has some limitations. First, this was a cross-sectional study and does not indicate the direction of causality in the observed insight–neuropsychology relationships. Second, the BCIS is a self-report measure and may not be very suitable for cognitively impaired patients.

In conclusion, the findings of this study demonstrate a modest association between the self-certainty, but not self-reflection, dimensions of cognitive insight and executive function in psychotic individuals which is not mediated by IQ. The self-certainty and self-reflectiveness dimensions of cognitive insight may have differential correlates, and possibly differential causes and mechanisms, in psychosis.

## Figures and Tables

**Table 1:** Descriptive statistics for insight measures and the correlations (Pearson's *r*) between cognitive and clinical insight.

	*N*	Mean (S.D.)
BCIS self-certainty	65	7.4 (3.8)
BCIS self-reflectiveness	65	14.1 (5.1)
PANSS G12	65	3.1 (1.4)
Birchwood insight scale		
Insight into symptoms	65	2.86 (1.31)
Insight into illness	65	2.52 (1.38)
Insight into treatment	65	3.21 (1.07)
Total	65	8.63 (2.96)
The expanded schedule of assessment of insight		
Insight into symptoms	57	3.86 (2.68)
Insight into illness	65	5.20 (2.53)
Insight into treatment	65	1.58 (0.70)
Insight into consequences	65	1.35 (1.01)
Total	57	12.30 (5.52)


Correlations	*N*	BCIS	BCIS
		Self-certainty	Self-reflectiveness
PANSS G12	65	0.375**	− 0.296*
Birchwood insight scale			
Insight into symptoms	65	− 0.420**	0.480**
Insight into illness	65	− 0.354**	0.276*
Insight into treatment	65	− 0.486**	0.169
Total	65	− 0.543**	0.400**
The expanded schedule of assessment of insight			
Insight into symptoms	57	− 0.418**	0.294*
Insight into illness	65	− 0.438**	0.391**
Insight into treatment	65	− 0.298*	0.181
Insight into consequences	65	− 0.321**	0.254*
Total	57	− 0.457**	0.428**

*P < 0.05; **P < 0.005.

**Table 2:** Descriptive statistics for neuropsychological variables.

Measure	*N*	Mean (S.D.)	Range
WASI IQ	63	101.42 (16.17)	70–133

*Executive function*			
WCST % perseverative errors	64	21.73 (14.61)	5–68
WCST % non-perseverative errors	64	19.78 (13.27)	1–63
WCST categories	64	3.55 (2.40)	0–6
Stroop interference score	64	− 1.47 (10.19)	− 18.8–46.8
Trails B-A score	64	82.89 (93.13)	2.3–495.4
Hayling profile score	64	5.38 (1.75)	1–9
Brixton profile score	65	5.06 (2.24)	1–10
BADS total score	62	15.98 (3.99)	2–22

*Verbal fluency*			
Phonological fluency (F, A, S)	64	36.69 (11.80)	5–62
Semantic fluency (categories)	64	40.77 (11.60)	18–75

*Sustained attention*			
CPT d′	64	0.86 (0.60)	− 0.1–2.4

*Memory*			
Hopkins verbal learning total correct	64	20.45 (5.65)	8–32

WASI – Wechsler Abbreviated Scale of Intelligence. WCST – Wisconsin Card Sorting Test. BADS – Behavioural Assessment of Dysexecutive Syndrome. CPT – Continuous Performance Test.

**Table 3:** Correlations between BCIS dimensions and the neuropsychological functioning.

Neuropsychological measure	*N*		BCIS	BCIS	PANSS G12 item
			Self-certainty	Self-reflectiveness	
WASI IQ	63	*r*	− 0.136	0.031	− 0.254

*Executive function*					
WCST % perseverative errors	64	rho^a^	− 0.037	0.087	0.126
WCST % non-perseverative errors	64	rho	− 0.195	− 0.046	0.055
WCST categories	64	rho	− 0.016	− 0.086	− 0.149
Stroop interference score^a^	64	rho	− 0.094	− 0.002	− 0.200
Trails B-A score^a^	64	rho	− 0.010	0.167	0.239
Hayling profile score	64	*r*	− 0.214	0.114	− 0.243
Brixton profile score	65	*r*	− 0.261^b^	0.052	− 0.220
BADS total score	62	*r*	− 0.375^c^	0.020	− 0.301^b^

*Verbal fluency*					
Phonological fluency (F, A, S)	64	*r*	− 0.102	0.072	− 0.260^b^
Semantic fluency (categories)	64	*r*	− 0.004	− 0.089	− 0.194

*Sustained attention*					
CPT d′	64	*r*	− 0.185	− 0.020	− 0.107

*Memory*					
Hopkins verbal learning test	64	r	− 0.116	0.040	− 0.111

WASI – Wechsler Abbreviated Scale of Intelligence. WCST – Wisconsin Card Sorting Test. BADS – Behavioural Assessment of Dysexecutive Syndrome. CPT – Continuous Performance Test.

a Spearman's rank correlations used where variable not normally distributed.

b *P* < 0.05.

c *P* < 0.00357 (Bonferroni-corrected alpha level).
